# Hospital-based violence prevention programmes in South Wales Emergency Departments: A process evaluation protocol

**DOI:** 10.1371/journal.pone.0293086

**Published:** 2023-10-25

**Authors:** Jordan Van Godwin, Graham Moore, David O’Reilly, Megan Hamilton, Niamh Clift, Simon C. Moore

**Affiliations:** 1 DECIPHer, School of Social Sciences, SPARK, Cardiff University, Cardiff, Wales, United Kingdom; 2 Wolfson Centre for Young People’s Mental Health, School of Social Sciences, SPARK, Cardiff University, Cardiff, Wales, United Kingdom; 3 South Wales Trauma Network, Swansea Bay University Health Board, Port Talbot, Wales, United Kingdom; 4 Academic Department of Military Surgery and Trauma, ICT Centre, Birmingham, United Kingdom; 5 Violence Research Group, School of Dentistry, Heath Park, Cardiff, Wales, United Kingdom; 6 Security, Crime and Intelligence Innovation Institute, SPARK, School of Dentistry, Cardiff University, Cardiff, Wales, United Kingdom; PLOS: Public Library of Science, UNITED KINGDOM

## Abstract

**Background:**

Addressing violence related harm is a global public health priority. While violence is primarily managed in the criminal justice system, healthcare supports and manages those injured by violence. Emergency Departments (EDs), the primary destination for those seriously injured, have emerged as a candidate location for violence prevention initiatives. There is limited evaluation of ED-based violence prevention, and a lack of guidance for the implementation and delivery of them. Nurse-led Violence Prevention Teams (VPTs) have been developed and implemented in two EDs in Wales, UK. This protocol describes methods used in the process evaluation of these VPTs.

**Aim:**

To understand how VPTs function, how they were implemented, and mechanisms of impact, as well as the exploration of wider contextual factors influencing their function.

**Methods:**

Adopting a critical realist approach and informed by the Medical Research Council (MRC) guidance for process evaluations, the process evaluation will employ qualitative methods to collect and analyse data: a scoping review of evidence of effectiveness that considers the causal mechanisms underpinning violence; a documentary analysis to determine operational considerations concerning the development, implementation and delivery of the VPTs; a descriptive analysis of routine ED data to characterise the prevalence of violence-related attendances in each ED; interviews with professional stakeholders (N = 60) from the violence prevention ecologies in which the VPTs are embedded.

**Discussion:**

This protocol outlines a process evaluation of a novel, nurse led violence prevention intervention. Findings will be used to inform policy makers’ decision making on whether and how VPTs should be used in practice in other EDs across the UK, and the extent that a single operational model should be adjusted to address the local characteristic of violence. To the authors knowledge, this is the first process evaluation of a UK-based, nurse led Emergency Department Violence Prevention Team.

**Trial registration:**

**Protocol registration** ISRCTN: 15286575. Registered 13^th^ March, 2023.

## Introduction

Violence is a global public health concern and cause of premature mortality [1] and morbidity, including enduring impacts on individuals and communities, and an increased risk of behavioural, emotional and physical health problems [2]. In the UK, violence presents as a significant burden on healthcare systems [3]. Across England and Wales, it is estimated that there are over 30 attendances to ED for assault-related injury per 100,000 population in a typical year, with over 3,000 attendances in the under 18 years of age group [3].

### Emergency departments and violence prevention

Hospital-based violence intervention programmes (HVIPs), based in Emergency Departments (EDs) [4–6], offer a unique contribution to violence prevention initiatives [7]. Individuals attending ED with violence-related injuries are at greater risk of reattendance in emergency care, police arrest and death [8–10], compared to patients attending ED with injuries unrelated to violence. HVIPS are able to capitalise on patients’ willingness to address contributing behaviours immediately following injury [11], and EDs are likely to encounter a subset of victims who may not be identified by the criminal justice system, or other statutory agencies involved with violence prevention [12]. Formative effectiveness evaluations suggest HVIPs in EDs can reduce revictimisation and patents’ involvement in the criminal justice system [7].

The development of HVIPs is aligned to UK Government initiatives that aim to promote a whole system multi-agency (WSMA) [13] approach to violence. For example, the 1998 Crime and Disorder Act requires the National Health Service (NHS), police, and local government to collaborate on crime reduction strategies; including data sharing to inform targeted responses to violence. Violence has been further prioritised in the Serious Violence Strategy [14]. These motivations are aligned with initiatives in the NHS promoting active population health management, digitally enabled whole-person care and evidence-based treatment pathways [15]. For example, the current NHS contract, which places requirements on health providers, now includes the Information Sharing to Tackle Violence (ISTV) Initial Standard Specification [16]. This requires monthly data on violence-related attendances to EDs to be collated, anonymised and shared with partners in local community safety partnerships.

While focus on the development of HVIPs in the UK continues to grow, to date, the evidence supporting them is primarily based on evaluations conducted in North American emergency healthcare systems [7]. With notable differences in emergency care between the UK and US, and in the aetiology of violence, the translation of HVIPs into a UK context undermines confidence of effectiveness. Despite this uncertainty and the lack of guidance for the implementation and delivery of these interventions in a UK context, HVIPs have been implemented. For example, in Scotland, the Scottish Violence Reduction Unit has placed Navigators in EDs [17]. The Navigators are volunteers who engage with patients (typically 25 years and younger) presenting with assault-related injuries. Navigators offer psychosocial support as well as referring patients to third sector organisations for further bespoke support. While the referral of patients for further support has the potential to promote enhanced quality and continuity of care, there remains a lack of evidence for the ED referral of children involved in both violence [18] and domestic violence [19,20]. Further, there is a dearth of robust studies focused on the referral of the most dominant population in respect of assault related injury [7], young men, and for victims of sexual violence [21].

### The South Wales violence prevention teams

An HVIP has been developed in South Wales, initially established in 2019 in an Urban ED setting which serves a large catchment area for a multicultural population covering both adult and paediatric patients. In 2021 the HVIP was expanded to a further Urban ED in South Wales, which serves a smaller population than the ED where the intervention was first developed but remains one of the busiest ED’s in Wales. These HVIPs, collectively referred to as Violence Prevention Teams (VPTs), are funded by the UK Home Office and Youth Endowment Fund (YEF) with the funding administered by the Office of the South Wales Police and Crime Commissioner (PCC). These VPTs are unique in the UK as they are nurse-led, whereas other HVIPs are primarily volunteer-led. The VPTs aim to identify patients who are aged ten years or older making an unscheduled attendance into ED. The care of children younger than 10 years of age is managed by the senior consultant paediatricians.

The VPTs identify eligible patients (either directly, or by referral from ED clinical colleagues), and work with them to identify any underlying vulnerabilities that contribute to their experience of violence. They offer support directly, and as appropriate, make referrals to statutory and third sector agencies with the facilities to provide short, medium or long-term support. The intervention is developed from knowledge of the causes and consequences of violence but works with patients with considerable heterogeneity surrounding the reasons for their exposure to violence. For those patients involved in risky behaviours and social practices which likely contributes to their ED attendance, for example those who are alcohol-dependant or misuse drugs, the VPTs are also informed by the concept of the ‘teachable moment.’ which has been defined as the timeframe immediately following a traumatic experience, where individuals are more receptive to both behavioural and attitudinal change [22]. An initial formative service evaluation conducted by the Violence Prevention Unit (VPU) and Public Health Wales (PHW) [23], and while only one of the VPTs was operational, developed a tailored theory of change for the VPT and framed it as a complex multi-component intervention comprising a core set of intended activities and functions which included work at multiple, interacting levels:

### Patient Level

- To provide advice and support to patients.
- To signpost and support patients’ engagement with other services that are appropriate to their level of need.

### Healthcare System Level

- Awareness-raising activities with the aim of ensuring that the VPTs become a fully embedded component in the emergency care systems in which they are situated.
- Training and upskilling of healthcare professionals to improve processes, including the identification of patients who have been exposed to violence, increase confidence in case reporting and data capture, ensuring safeguarding procedures are followed and improve the patient referral process into the VPT.
- To formalise the assessment of risk and need for patients with assault related injury.

### Broader System Level

- Working in partnership with statutory services such as Social Services and the Police as well as Third Sector agencies to ensure patients receive adequate support and needs assessment [23].

While the population for VPTs is primarily ED patients attending ED with injuries arising through violence, the broader influence of VPTs (based on the components outlined above) means that their activities can influence the provision of care in EDs and outpatient services (through upskilling clinical staff and improving referral processes for their patients). They can also influence the broader violence prevention ecology through the improved ascertainment of violence ‘hotspots and information sharing with and patient referral to, third sector agencies.

It is important to note however, that the formative process evaluation [23] could only report on one of the VPTs as the other VPT was in the process of being set-up. Therefore, while the core components of one of the VPTs have been identified and reported, no process evaluation data currently exists for the more recently established VPT, and there is no information on the similarities and differences between the two iterations of this intervention. As a result, data concerning the generalisability of the VPT model into new contexts as well as any adaptation required remains missing.

### Rationale for the process evaluation

Despite the emergence of HVIPs as a public health response to violence-related emergency healthcare utilisation [6,24], there has been no process evaluation of this public health approach and there is therefore a lack of guidance for the implementation and delivery of these interventions in the UK. This is particularly important as the VPT intervention had been identified as a complex intervention [23], with ‘several interacting components’ and they are implemented in and interact with already complex systems [25] the context of which includes wider healthcare services, criminal justice systems and third sector organisations. Process evaluation in these circumstances therefore becomes essential as it provides the necessary format to explore the implementation, causal mechanisms and contextual influences associated with complex interventions [25].

The aim of this paper is to describe the protocol for a process evaluation of the VPTs in South Wales EDs. An earlier version of the protocol for this process evaluation has also been published on the Youth Endowment Fund website [26] and the study has been registered with the International Traditional Medicine Clinical Trial Registry (ISRCTN: 15286575).

### Aims and research questions

Adopting a critical realist approach, the overarching aim of this work is to conduct a process evaluation following the MRC guidance for process evaluation. This approach to process evaluation involves:

- determining the quantity and quality of the implementation,
- describing the mechanisms through which an intervention (if delivered as intended) may activate change, and
- describing the contextual conditions perceived as facilitating or constraining the success of an intervention [25,27].

Recent guidance for adapting interventions to new contexts [28], will also be drawn upon to inform understandings of how the intervention has been adapted for a new setting while maintaining consistency with the core functions of the VPT model. This will also be used to inform considerations of to what other contexts the intervention might be transferred, and therefore be subject to further adaptation. The VPT function will be further contrasted with what is known to work for populations exposed to violence and determine the extent that the VPT makes an evidenced-based contribution to health service delivery.

In order to achieve this a series of research questions were co-produced with stakeholders, including partners from the UK Home Office; the Violence Prevention Unit (VPU; which commissioned the VPTs) and Public Health Wales (PHW). This formative work identified nine primary and two secondary research questions:

1. To what extent have VPT’s become embedded within broader hospital systems?
2. To what extent do implementers adhere to the intended delivery model?
3. How much of the intended intervention has been delivered?
4. How well are the different components of the intervention being delivered?
5. To what extent does the intervention reach the entirety of assault-related ED attendances?
6. To what extent do patients engage with the intervention?
7. How were in-hospital referral pathways developed for patients, and to what extent were patients supported across institutional transitions?
8. What is the perceived need for and benefit of the intervention amongst the implementers and related stakeholders?
9. What strategies and practices are used to support high quality implementation?

Our secondary research questions are:

1. What adaptations were undertaken to use the VPT model in a new context and why?
2. What are stakeholders’ views on the types of setting to which the model is likely to be more or less transferable?

## Materials and methods

In order to address the research questions, the following methods will be employed: a scoping review, a documentary analysis, semi-structured interviews and an analysis of routine data. Table 1 provides an overview of the research questions addressed by each method.

**Table 1:** Research questions addressed by each method.

Method	Research Question
**Scoping Review**	9. What strategies and practices are used to support high quality implementation?
**Document Analysis**	1.To what extent have VPT’s become embedded within broader hospital systems? 2. To what extent do implementers adhere to the intended delivery model? 3. How much of the intended intervention has been delivered? 4. How well are the different components of the intervention being delivered? 5. To what extent does the intervention reach the entirety of assault-related ED attendances? 6. To what extent do patients engage with the intervention? 7. How were in-hospital referral pathways developed for patients, and to what extent were patients supported across institutional transitions? 8. What is the perceived need for and benefit of the intervention amongst the implementers and related stakeholders? 9. What strategies and practices are used to support high quality implementation? 10. What adaptations were undertaken to use the VPT model in a new context and why?
**Routine data analysis**	5. To what extent does the intervention reach the entirety of assault-related ED attendances?
**Interviews**	1. To what extent have VPT’s become embedded within broader hospital systems? 2. To what extent do implementers adhere to the intended delivery model? 3. How much of the intended intervention has been delivered? 4. How well are the different components of the intervention being delivered? 5. To what extent does the intervention reach the entirety of assault-related ED attendances? 6. To what extent do patients engage with the intervention? 7. How were in-hospital referral pathways developed for patients, and to what extent were patients supported across institutional transitions? 8. What is the perceived need for and benefit of the intervention amongst the implementers and related stakeholders? 9. What strategies and practices are used to support high quality implementation? 10. What adaptations were undertaken to use the VPT model in a new context and why? 11. What are stakeholders’ views on the types of setting to which the model is likely to be more or less transferable?

### Scoping review

Following the Preferred Reporting Items for Systematic Reviews and Meta-analysis Protocols Scoping Review (PRISMA-ScR) [29], a scoping review of emergency care-based interventions for those who experience violence and the underpinning causal mechanisms of violence informing interventions will be conducted. The review will focus on what emergency care interventions work for those experiencing violence as well as how they work and in what context, including what is required from an intervention implementation perspective. The review will also explore the causal mechanisms, and nature of predisposing characteristics upon which attendance in emergency care with an assault-related injury is predicated, as this can be essential in informing for whom, how and in what context interventions can be delivered.

The search strategy for this scoping review study will involve electronic databases including PubMed, Web of Knowledge, Science Direct, EBSCOHost (PubMed, CINAHL with Full Text, MEDLINE), Google Scholar, BioMed Central and the Cochrane Library. Articles will also be searched through a “cited by” search as well as citations included in the reference lists of included articles. Reference sections in each document identified as relevant will also be reviewed for further relevant research. In academic papers, articles and websites, lists of articles citing documents identified as relevant will also be reviewed. Keyword searches will be used, and two reviewers will be screening titles, abstracts and full articles. Thematic analysis will be employed to present a narrative account of the review.

### Documentary analysis

Documentary analyses will focus on materials including role descriptors for members of each VPT and hospital Standard Operating Procedures, particularly those focused on managing assault related injuries. These will enable an initial understanding of the extent to which VPTs are achieving the aim of establishing a presence within the hospital, health board and across wider agencies. These will be subjected to a content analysis, capturing the number and qualitative nature of mentions of VPTs, and summarising how their roles are represented, within and between the VPTs across the different EDs. We will further assess the extent to which VPTs are represented on and participate in broader violence prevention initiatives within and external to the health service, such as cross-partner initiatives in local government such as Community Violence Prevention Teams.

### Semi-structured interviews

Participants will be recruited purposefully across the violence ecology, stratified by sector (e.g., police, health) and responsibility (e.g., practitioner, decision maker, advisor, commissioner). Their identities are known to the broader research team, intervention partners, and available through additional snowball sampling. Interviews will be conducted between January-September, 2023, with up to 30 agents across each of the two local violence prevention ecologies (N = 60). Interviews will be used to explore the extent to which VPTs have or have not become embedded within these systems. Interviews will begin with members of the VPTs themselves, and of their respective local health boards. A semi-structured interview guide will be used during interviews to situate VPTs within the broader ecology of practice and describe the inter-relationships between partners. Interviews will also explore (but will not be limited to): how ED data is captured in patient management systems, by whom, at what stages of the patient pathway; any classification (formal or informal) of patients as having an assault related injury; how VPT augments existing job roles and expectations; partner requirements for VPT activity data and what, if any, opportunities for these data to inform violence reduction initiatives exist; whether there are any legal, technical, financial or contextual considerations for interagency cooperation.

The interviews will complement the documentary analysis by promoting a detailed understanding of the strategies adopted for establishing the presence of the VPTs, and the extent to which role descriptors capture the work of the VPTs in sufficient detail to enable the essence of these roles to be replicated across contexts. Interview recruitment will continue until either data saturation is reached or 30 interviews have been completed in each site. Qualitative data will be transcribed verbatim and analysed using NVivo 12 software [30].

#### Patients and service users

The primary focus of this evaluation is the context and systems in which the evaluation is embedded. While including qualitative data from patients exposed to the intervention may have provided additional insights, obtaining necessary ethical approvals and recruiting young people to interviews was not possible within the time and resources available. Instead, we will conduct the following in order to cover some of the questions patient input could have provided:

- Collate summary statistics on patient engagement collected by the VPTs.
- Through the interviews, extensively question key stakeholders on this to ascertain their perspective on patient engagement and experiences.
- Conduct PPI work with young people to gather their perspectives on the intervention and perceived engagement challenges and opportunities.

#### Routine data

Routine healthcare data from the Emergency Department Data Set (EDDS), to characterise the nature and incidence of assault-related injury attendances at both EDs. These data are anonymised, aggregate, and subject to existing approvals between the research team and the data controllers. Further, public open police data, which provide anonymous incident detail for crimes across England and Wales, will be used to characterise violence against the person incidents involving the police in the catchment area of the two EDs. Data will be accessed between June-September 2023.

Existing routine data will inform the qualitative research by capturing possible reasons for variation in intervention design to meet local conditions, for example ethnic, gender or age variations that might warrant different approaches across the two EDs (e.g., translators for non-English speakers, liaison with independent domestic violence advocates, involvement of youth social services).

#### Public and patient involvement

The research questions and design of our process evaluation was coproduced with key stakeholders from the VPU, Public Health Wales and the Home Office. Further, over the course of the process evaluation, we aim to conduct four sessions with groups of young people. To date, two sessions have been conducted and a further two are scheduled to take place at the end of the study in November 2023. The first sessions explored young people’s views on the VPT intervention and its approach, and their views on the approach of the process evaluation, including an exploration of young people’s views on what research questions should be asked of VPT staff and the wider group of professionals. The aim of the follow-up sessions will be to report back to the group of how we used their initial feedback to inform and influence the evaluation, to report the findings of the evaluation and to ask for their views on the results, ideas for dissemination and future research ideas to build on this work.

### Ethical approval and consent to participate

This study was given a favourable ethical opinion on 25th November 2022 by the Cardiff University School of Dentistry Research Ethics Committee (REF: DSREC/2213a). Informed consent will be required for all interview participants.

### Evaluation frameworks

The process evaluation is informed by a critical realist approach, incorporating participants’ subjective experience, elucidated using interviews, while also recognising and identifying the social systems (i.e., the emergency department, broader healthcare system and violence prevention ecology) in which the behaviour occurs, and which simultaneously acts to enable and constrain social practices [31]. It uses the MRC guidance for process evaluation which emphasises the importance of implementation, mechanisms, and context (and is itself influenced by critical realism informed evaluation approaches) [25]. Emerging frameworks for adapting interventions in new contexts is also utilised [28,32] as when considering intervention adaptation, decisions will be driven by considering the contextual similarity and differences between the two EDs and environs, and the contextual conditions necessary for the intervention(s) to function as intended.

### Qualitative data analysis

**Scoping review analysis.** Thematic analysis will be employed to present the narrative account of the review.

**Documentary analysis.** Documents will be subjected to a thematic content analysis, capturing the number and qualitative nature of mentions of VPTs, and summarising how their roles are represented, within and between the two EDs.

**Semi-structured interviews.** Adopting a critical realist approach, thematic analyses [33] of the interview data will be conducted and will include both deductive and inductive elements [34] to support the exploratory and structured nature of this evaluation. This process will involve open reading to engage with participant understanding and experience [35] and will explore the implementation of the VPTs and its relationship with the violence ecology. A programme model for each site will be developed in order to understand the micro-, meso- and macro-organisational and policy contexts of the VPT (e.g. barriers, governance, funding, strategic partnerships, data systems, acceptability) so the VPTs can be situated within the broader ecology of practice and describe the inter-relationships between partners and sectors.

### Quantitative data analysis

Descriptive analyses of anonymised ED data will be conducted. The routine health and police data will be analysed to characterise the distribution and nature of presenting conditions of unscheduled attendances in each ED. Existing routine data does not inform the qualitative work, beyond capturing possible reasons for variation in intervention design to meet local conditions, for example ethnic, gender or age variations that might warrant different approaches across the two EDs (e.g., translators for non-English speakers, liaison with independent domestic violence advocates, involvement of youth social services).

### Mixed-method triangulation

Thematic analysis will be employed for the scoping review, document analysis and interview data to facilitate both the structured (research questions and existing frameworks) and exploratory elements of our evaluation and ensure consistency in both the analytical and reporting process. Triangulation of the qualitative and quantitative data will be conducted to explore similarities and differences between these data to enhance our understanding of the implementation and delivery of the VPTs.

## Discussion

This paper outlines the protocol for a process evaluation of the VPTs in South Wales, a nurse-led service based in two EDs that identify and support patients attending ED with assault-related injury, have a broader pedagogical role that increases awareness of these patients’ needs in the ED, and conduct awareness raising activities so that VPTs become a fully embedded component in the emergency care system. The aim of the process evaluation is to understand the functioning of the existing VPT intervention model through the examination of implementation, impact mechanisms, and context by utilising qualitative interviews, document analysis and examining routine data. A focus on context will also promote an understanding of questions regarding transferability and local adaptation. In publishing this protocol, the authors seek to emphasise the importance of a process evaluation when evaluating complex hospital-based interventions. To the authors’ knowledge this will be the first process evaluation of a UK-based, ED-based, nurse-led violence prevention intervention, thereby contributing simultaneously to the growing literature on process evaluation and hospital-based violence prevention interventions.

It is essential that the VPT is understood with reference to the underpinning knowledge base. Across a population, the likelihood of violence varies systematically. Individual experiences, including direct experience or observational learning, increases the likelihood that someone will engage in violence [36–39]. There are also strong socio-economic and demographic correlates [40–42]: male, socio-economically deprived individuals are more likely to experience violence. The context, or environment, in which people find themselves will also motivate violence, notably areas in which alcohol is sold and consumed [43,44], as well as domestic and workplace environments. While the population for VPTs is primarily ED patients attending ED with injuries arising through violence, the broader influence of VPTs means that their activities have the potential to influence the provision of care in EDs (through upskilling clinical staff and improving referral processes for their patients] and the broader ecology through the improved ascertainment of violence “hotspots”. However, there is limited evidence to date which describes whether or how this has taken place. To address this, the process evaluation will, through the interview process, recruit the stakeholders necessary to understand whether and how the knowledge generated by the VPTs is disseminated and utilised. There is also currently limited evidence available to inform policy makers’ decision making on whether and how the VPT intervention should be used in respect of other ED departments across the UK. This process evaluation aims to address this and has been designed to generate a detailed understanding of the design, implementation, and impact of the VPTs through interviews with professional stakeholders across the violence prevention system in both sites. The findings from this process evaluation could provide significant support to other healthcare systems looking to adopt a similar violence prevention model as well as offering an understanding of how these types of interventions can influence practice across the wider violence prevention ecology.

## Abbreviations

ARA: Assault Related Injury
ED: Emergency Department
HVIPs: Hospital-based violence intervention pro
NHS: National Health Service
PCC: Police and Crime Commissioner’s Office
PHW: Public Health Wales
SWP: South Wales Police
UHB: University Health Board
VPT: Violence Prevention Team
VPU: Violence Prevention Unit
VRU: Violence Reduction Unit
YEF: Youth Endowment Fund
